# The 2022 Performance Report of the *Malaysian Journal of Medical Sciences*

**DOI:** 10.21315/mjms2023.30.6.1

**Published:** 2023-12-19

**Authors:** Zulkapli Nour Azimah, Jafri Malin Abdullah

**Affiliations:** 1 Malaysian Journal of Medical Sciences, Universiti Sains Malaysia, Pulau Pinang, Malaysia; 2 Malaysian Journal of Medical Sciences, Universiti Sains Malaysia, Kelantan, Malaysia

**Keywords:** journal performance, submission trend, journal impact

## Abstract

This is an annual performance report of *Malaysian Journal of Medical Sciences* (MJMS) for the year 2022. This report presents data pertaining manuscript submissions to MJMS and its performances are presented in various form of views.

## Introduction

The Editorial Office presents report on the performance of the *Malaysian Journal of Medical Sciences* (MJMS) throughout the year 2022. Data of new manuscript submissions and other information were extracted from the manuscript submission platform and other databases.

### Submission Trend of New Manuscripts

**Figure 1:**
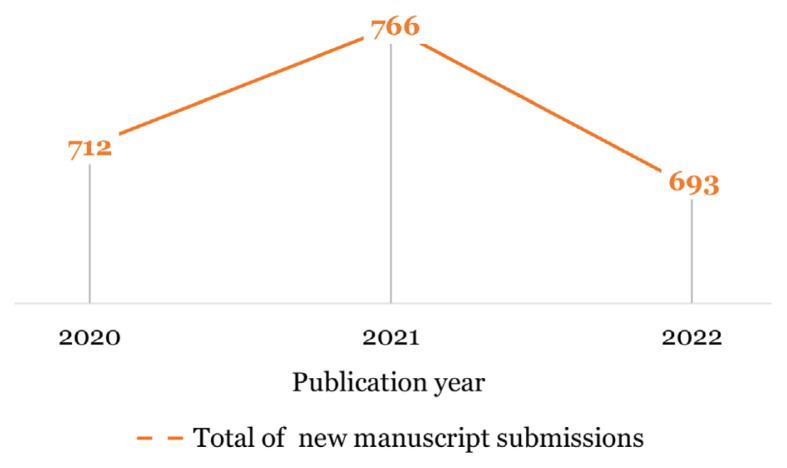
Total submissions of new manuscripts in 2020–2022 Source: https://mc.manuscriptcentral.com/maljms

Over the years, MJMS received an overwhelming number of manuscript submissions. However, the submission of new manuscripts to MJMS in 2022 had declined 9.5% compared to the preceding years (1, 2). Figure 1 shows the submission trend throughout the COVID-19 era in 2020 until 2022. This declination is perceived to be the situation of returning back to the norm after an unexpected enormous number of submissions happened during the COVID-19 outbreak throughout the years 2020 and 2021.

### Submission Pattern by Manuscript Type

As usual, Original Article manuscript is the type of manuscript mostly submitted to MJMS followed by Review Article manuscript. This proves that there are a great number of research works being done on the new topic of interests that eventually contributed to the original findings, results and data which then being presented and authored in the Original Article manuscript.

**Figure 2:**
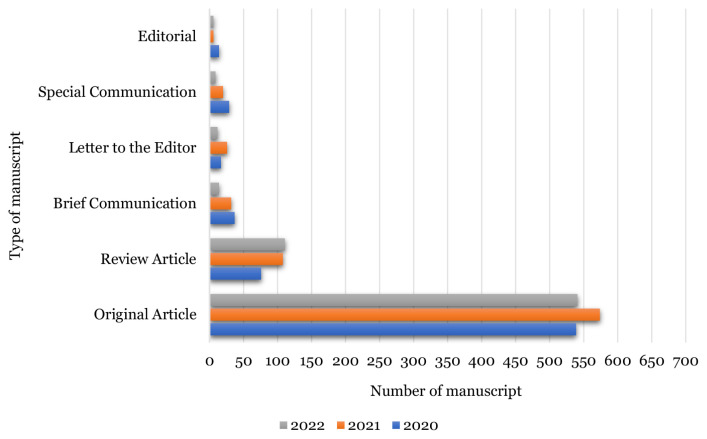
Submissions trend by manuscript type in 2020–2022 Source: https://mc.manuscriptcentral.com/maljms

As shown in Figure 2, the submission of Original Article manuscripts had decreased by 5.8% in 2022 compared to submissions in 2021. The decrement of submission also happened to other manuscript types except Review Articles where there was a slight increment of 2.8% in 2022 compared to the submission in 2021.

### Submission Pattern of New Manuscripts in Percentage by Year and Month

**Figure 3:**
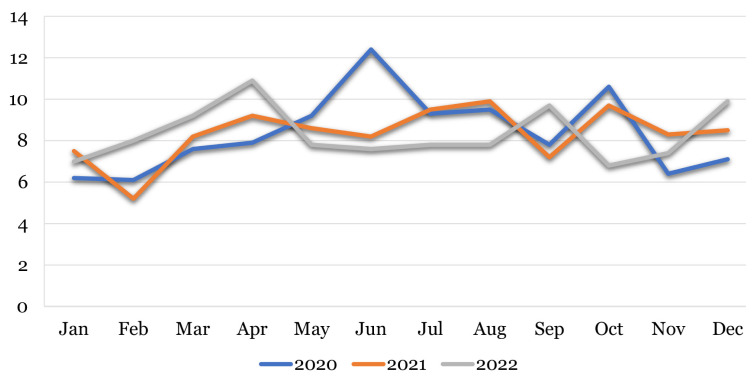
Submission patterns of new manuscripts in percentage in 2020–2023 Source: https://mc.manuscriptcentral.com/maljms

In regard to the percentage of new manuscript submissions by year and month, the highest was 10.9% in April compared to other months in 2022 and 2021. The comparison of submission patterns by month and year of the preceding years is shown in Figure 3.

### Contributions by Country

Manuscript contribution to MJMS was exclusively led by Malaysia. Table 1 shows the acceptance of manuscript from Malaysia and other main countries in the most three recent years between 1 January 2020 and 31 December 2022. Despite the huge number of manuscripts respectively submitted by each country, the percentages of rejection are also considered high.

Data extracted from the system shows that the rejection rate for a total of 682 manuscripts with a decision dated in 2022 was 85.3%, 79.5% (606 manuscripts) in 2020 and 80.1% (700 manuscripts) in 2021.

### Contribution by Institution

**Figure 4:**
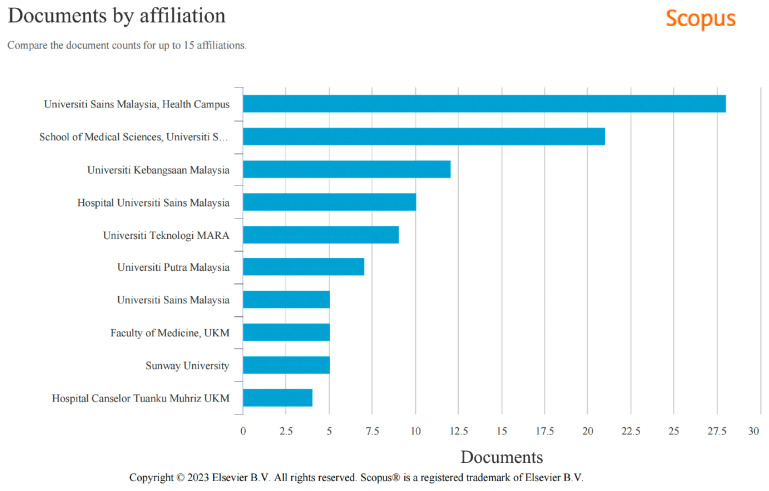
Institutions as major contributors to MJMS in 2022 (3)

Figure 4 shows the comparison of manuscript contribution counts in 2022 among local higher institutions in Malaysia. This data was generated based on the author’s input during manuscript submission to the journal’s system.

The School of Medical Sciences Health Campus of Universiti Sains Malaysia has dominated the list followed by Universiti Kebangsaan Malaysia, Hospital Universiti Sains Malaysia, Universiti Teknologi MARA, Universiti Putra Malaysia and the Pulau Pinang Campus of Universiti Sains Malaysia.

### Journal Impact Factor

The JIF of MJMS for the year 2022 is 1.4. This metric is based on the latest Journal Citation Reports (2023) by Clarivate Analytics and calculated from data indexed on the Web of Science Core Collection where 168 citable manuscript titles were the contributing items to this JIF score.

The JIF is calculated using the following metrics:


$$\begin{array}{l} \frac{\begin{array}{c} \text{Citations in }2022\;\text{to items published in}\\ 2020\;(165)+2021\;(73) \end{array}}{\begin{array}{c} \text{Number of citable items in}\\ 2020\;(85)+2021\;(83) \end{array}}\\ =\frac{238}{168}\\ =1.4 \end{array}$$

The JIF reflects the annual average number of citations to recent articles published in that certain journal. Hence, publication in journals with higher JIF may have higher chance of being cited as compared to publications in journals with relatively low JIF.

#### Rank by Journal Impact Factor

MJMS and other journals indexed in the Emerging Sources Citation Index (ESCI) are receiving a JIF for the first time in June 2023. Therefore, they will not receive ranks, quartiles or percentile until the release of 2023 data in June 2024.

### Quartile in Scopus Indexation

Scopus assesses journals using a journal quality clustering system known as Quartile (Q). Based on the data provided by Scopus, the highest percentile gained by MJMS was 61% (in rank 322/830) in category General Medicine with CiteScore 2.6 in 2022, 68% (in rank 258/826) with CiteScore 2.5 in 2021 and 65% (in rank 273/793) with CiteScore 2.0 in 2020. These percentiles indicated that MJMS is included as Quartile 3 category in the quartile ranking (5).

## Figures and Tables

**Table 1:** Main contributors and accepted manuscripts with a decision date between 1 January 2020 and 31 December 2022

Country/Region	Total manuscript	Acceptance (%)
Malaysia	749	34.05
Indonesia	279	9.32
India	268	5.60
Pakistan	88	6.82
Saudi Arabia	65	7.69
Turkey	46	4.35
Thailand	22	27.27
Vietnam	16	25.00

Source: https://mc.manuscriptcentral.com/maljms
